# External Validation of the Cancer of the Prostate Risk Assessment Postsurgical Score for Prediction of Disease Recurrence after Radical Prostatectomy

**DOI:** 10.1155/2016/8639041

**Published:** 2016-10-19

**Authors:** Taha Numan Yıkılmaz, Erdem Öztürk, Eşref Oğuz Güven, Halil Başar

**Affiliations:** Department of Urology, Ankara Dr. Abdurrahman Yurtaslan Oncology Training and Research Hospital, Ankara, Turkey

## Abstract

*Objective*. The cancer of the prostate risk assessment (CAPRA-S) postsurgical score predicts recurrence, metastasis, and cancer-specific survival after radical prostatectomy (RP). We evaluated the relation between CAPRA-S score and biochemical recurrence (BCR) in prostate cancer after RP in our clinic.* Materials and Methods*. This study was performed on 203 patients with prostate carcinoma who underwent open RP and regional lymph node dissection in our clinic between 2008 and 2013. We calculated the CAPRA-S scores including prostate-specific antigen (PSA) at diagnosis, pathology Gleason score, surgical margin, seminal vesicle invasion, extracapsular extension, and lymph node involvement. The patients were divided into 3 risk groups (low, intermediate, and high risk) according to risk scores.* Results*. Recurrence occurred in 17.8% of the patients (36 patients out of 203 patients) with a median of 11.7-month follow-up. The average recurrence-free survival time is 44.6 months. Surgical margin invasion and seminal vesicle invasion significantly correlated with BCR especially in high risk group (11 and 13 of 15 patients, *p* < 0.05, resp.).* Conclusion*. CAPRA-S score can be easily calculated and it is useful in clinical practice in order to timely propose adjuvant therapies after surgery.

## 1. Introduction

Prostate cancer is the most common noncutaneous malignancy. In 2014, 233000 new cases were diagnosed and 29480 cancer-specific deaths were estimated [1]. A radical prostatectomy is the common primary treatment of clinical localized prostate cancer (Pca) [2]. Although radical prostatectomy is an effective treatment of localized prostate cancer, about one-third of patients have biochemical recurrence (BCR) after radical prostatectomy (RP). Biochemical recurrence is detected by prostate-specific antigen (PSA) elevation after operation [3]. Fifty-two percent of patients with BCR have been shown to have extraprostatic extension [4]. Risk classification is important for selecting the proper treatment; thus some nomograms have been developed in different study cohorts. In 2005, UCSF (University California-San Francisco) proposed a nomogram known as Cancer of the Prostate Risk Assessment (CAPRA), a pretreatment score based on patient age, PSA, biopsy Gleason score, clinical stage, and percent of positive biopsy cores [5]. In 2011 the same group revised the score system and named CAPRA-S score. Pathology findings like pathologic Gleason score, surgical margin, extracapsular extension, seminal vesicle invasion, and lymph node involvement were added to the new score system [6]. This new system is validated by various studies and confirmed BCR prediction [4, 7–9]. In this study we examine the validity of the CAPRA-S score in our institution.

## 2. Patients and Methods

This study was performed on 203 patients with Pca who had open RP and regional lymph node dissection in our clinic between 2008 and 2013. Data were collected retrospectively. Among the 241 patients identified, patients who received neoadjuvant treatment and no information about data prevented us from calculating CAPRA-S, thus leaving 203 men available for final analysis. We calculated the CAPRA-S scores as described by Cooperberg et al. (Table 1) [6]. This score has 6 variables including PSA at diagnosis, pathology Gleason score, surgical margin (SM), seminal vesicle invasion (SVI), extracapsular extension (ECE), and lymph node involvement (LNI). The CAPRA-S score is calculated using the points reported in Table 1. The patients were divided into 3 risk groups (low, intermediate, and high risk) according to risk scores. Low risk groups were between 0 and 2 points, intermediate groups were between 3 and 5 points, and high risk groups were above 6 points of score sum (Table 2). Biochemical recurrence was defined as increasing of PSA ≥ 0.2 ng/mL following RP.

CAPRA-S score as predicting BCR was analyzed by Cox proportional hazards regression and Kaplan-Meier analysis by use of SPSS ver. 23.0 (IBM Co., Armonk, NY, USA). Harrell's concordance index (c-index) was calculated to evaluate the 3-year prediction probabilities of CAPRA-S score and three-risk level model. Informed consent was not obtained due to retrospective design of study.

## 3. Results

**Figure 1:**
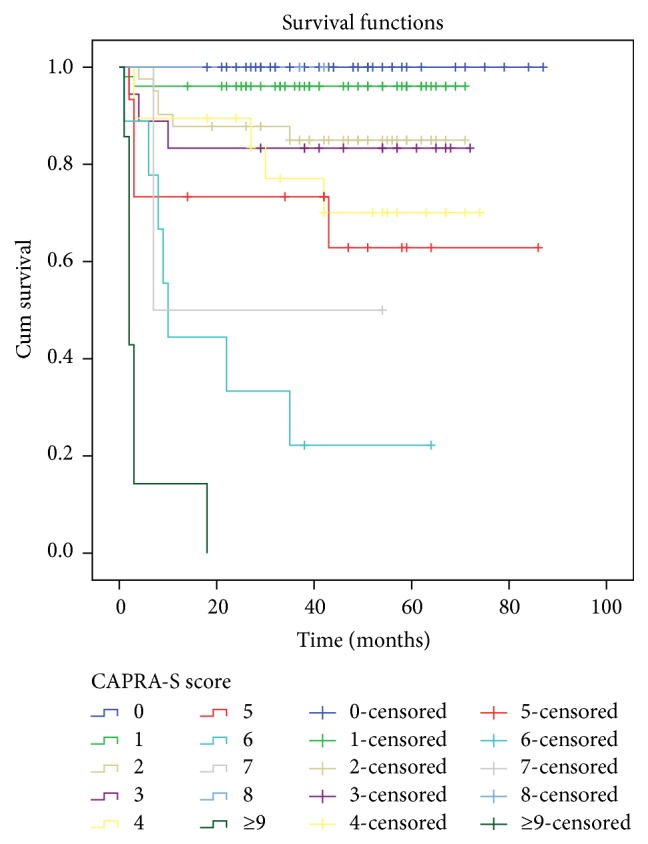
Biochemical recurrence after radical prostatectomy, stratified by grouped CAPRA-S scores using Kaplan-Meier curves.

**Figure 2:**
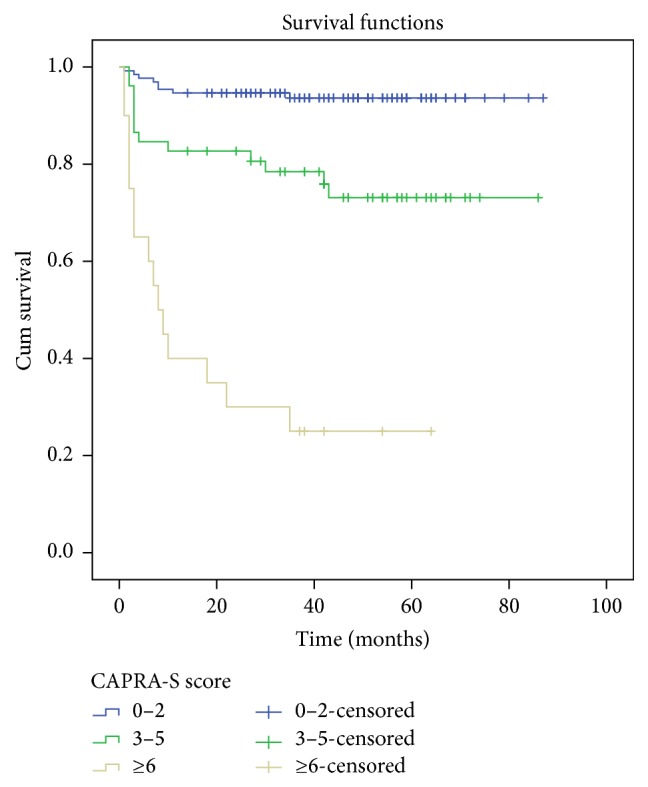
Biochemical recurrence by CAPRA-S risk groups (each *p* < 0.001).

The mean age of patients were 64.5 years (ranging from 51 to 84 years) and mean PSA values were 9.6 ng/mL (range 2.9–42 ng/mL). Pathological features in CAPRA-S scoring system were shown in Table 1. Patients were divided into 3 groups according to CAPRA-S. There were 131 (64.5%), 52 (25.6%), and 20 (9.9%) patients in low, intermediate, and high risk groups, respectively. Each score group (beginning from 0 to >9) is as follows (patients, %): 39 (19.2%); 51 (25.1%); 41 (20.1%); 18 (8.8%); 19 (9.3%); 15 (7.3%); 9 (4.4%); 2 (0.9%); 2 (0.9%); and 7 (3.5%). Recurrence occurred in 17.8% of the patients (i.e., 36 patients out of 203 patients) with a median of 11.7-months follow-up. The average recurrence-free survival time is 44.6 months in all patients. We showed the relationship between BCR and CAPRA-S scores in Figures 1 and 2. When we investigated each group, low, intermediate, and high risk groups, BCR was determined as 8 of 131 (6.1%), 13 of 52 (25%), and 15 of 20 patients (75%), respectively (*p* < 0.05, Figure 2). Surgical margin invasion was significantly correlated with BCR, especially in the high risk group (13 of 15 patients, *p* < 0.05). Biochemical recurrence was observed in 18 of 38 patients (47.3%) with positive SMI which was statistically significant (*p* < 0.05). Extracapsular extension did not show any statistically significant correlation with BCR (13 of 41 patients, *p* > 0.05). However in the high risk group 11 of 15 patients (73.3%) was seen with BCR (*p* < 0.05). Biochemical recurrence was observed in 8 of 13 patients (62%) with positive SVI which has statistically significant positive correlation (*p* < 0.05). Surgical margin and seminal vesicle invasion showed statistical significance with a hazard ratio (HR) of 1.60 (*p* = 0.035) and 1.49 (*p* = 0.041), respectively. There was no statistically significant correlation for other variables. These variables were inserted into a Cox proportional hazards regression model (Table 4). As a result, the risk of BCR increased with high scores.

The CAPRA-S score has high concordance value and we have just determined three-risk level model in 3-year BCR-free probabilities. The c-index of each CAPRA-S score group for the 3- year BCR-free probabilities rate was 0.82 (*p* < 0.05). When we investigated in three-risk level model, c-index score was 0.78 (Table 3).

## 4. Discussion

Radical prostatectomy is a standard treatment of localized prostate cancer and one-third of prostate cancer patients in US undergo radical prostatectomy [10]. This operation can be performed open, laparoscopic, and robotic. In all surgical ways BCR was a common problem in postoperative term. Despite primary treatment of localized prostate cancer 20–30% of patients experience a BCR [11, 12]. We found 17.8% BCR in our study and obtained a similar result with the previous studies in the literature [9]. From Shared Equal Access Regional Cancer Hospital (SEARCH) database Punnen et al. calculated a ratio of 34.3% [7].

Postoperative PSA levels can help us to estimate BCR, but PSA is not enough to identify BCR in some cases [13, 14]. Many researchers have tried to develop a nomogram to overcome this challenge [15]. Cooperberg et al. developed a nomogram named CAPRA score in 2005. The parameters in CAPRA score were preoperative PSA, biopsy Gleason score, clinical T stage, percent of positive biopsies, and age at diagnosis [5]. This score was validated in the US and European studies and it is demonstrated that CAPRA score is compared to other nomograms [4, 16, 17]. In 2011 Cooperberg et al. described the CAPRA-S score, including PSA, SMI, SVI, ECE, LNI, and Gleason score, that predicted BCR better than CAPRA score [6].

CAPRA-S score has not been validated in US and Europe until last year. Punnen et al. studied in 2670 patients in 2014 and CAPRA-S score was validated in US by this study [7]. This study showed significant correlation between CAPRA-S and BCR. Tilki et al. evaluated the CAPRA-S score in 14532 patients who underwent RP in Martini-Clinic and compared with CaPSURE data set [9]. They found the relationship between high risk scores in CAPRA-S with BCR and metastasis. This study reported the first independent validation study of CAPRA-S in Europe. We performed the CAPRA-S score of 203 patients who underwent RP in prostate cancer. We found significant correlation with high risk scores and BCR as stated in the literature [4, 8, 9].

The CAPRA-S scoring system has a high value of c-index in RP [5]. Concordance value was found as 0.77 in CAPRA-S score developed by Cooperberg. Later Seong and Punnen reached similar levels and presented their results [7, 18]. In 2013 Seong et al. reported that the c-index of CAPRA-S score for the BCR-free probabilities was 0.80 in 134 Korean patients with Pca and one year later Seo reviewed c-indexes as high as 0.80 in 130 Korean patients [4, 18]. The c-index was found as 0.73 for predicting BCR from multi institutions in US by Punnen et al. [7].

Tilki et al. who have the largest series about CAPRA-S after RP in the literature found similar results. As a result of this study, CAPRA-S c-index which predicts BCR was 0.80. Also CAPRA-S c-index predicting metastasis and mortality was 0.85 and 0.88, respectively [9].

In our study c-index for 3 years BCR-free probabilities was 0.82 and 0.78, when considering single patient scores or the three-risk level grouping, respectively. These results were shown to be helpful in predicting postoperative BCR in our patients based on their CAPRA-S score. When we compared the results in our study to the ones presented by Tilki and Punnen who have large patients series about CAPRA-S, there are some similarities and differences. Punnen et al. have used SEARCH database in their study and recurrence occurred in 34.3% of patients at a median of 14 months. They determined association between BCR with increasing risk according to Kaplan-Meier curves as our study. CAPRA-S c-index scores were found to be 0.73 and 0.82, respectively, in Punnen et al.'s and our study. However Punnen et al. studied 5-year BCR-free survival and association between CAPRA-S score and metastasis and mortality in different results from our study [7]. When we examined Tilki et al.'s study, similar results were obtained with Punnen et al. [9]. Similar results were found in our study with two large studies except some limitations.

The limitations in our study were small number of patients, retrospective design, limited follow-up period, and low percentage (9.9%) of high risk patients. The low percentage of high risk patients is due to the fact that we do not routinely suggest prostatectomy to these kinds of patients. In addition we did not evaluate 5-year BCR-free survival and progression-free survival due to limited follow-up period. Even so, we demonstrated possible usefulness of the CAPRA-S score in management of patients who underwent RP.

## 5. Conclusion

Although BCR does not correlate with cancer-specific survival, adjuvant therapy should be given to patients with poor pathology results. It is difficult to predict recurrence; therefore nomograms were developed to estimate BCR in prostate cancer. CAPRA-S score can be easily calculated and used in clinical practice without any loss of time. We have no information on use of the score to predict metastasis and mortality after surgery in our population; however it is mentioned that the CAPRA-S score system may be useful in predicting metastasis and mortality in the literature. It is useful for predicting BCR, metastasis, and mortality after surgery with a c-index of greater than 0.80. It can be used to decide on adjuvant treatment after surgery.

## Figures and Tables

**Table 1:** Distribution of data according to CAPRA-S score.

Variable	Level	Points	Number (%)
PSA (ng/mL)	0–6	0	69 (33.9)
	6.01–10	1	88 (43.4)
	10.01–20	2	38 (18.7)
	>20	3	8 (4)

Surgical margin	Negative	0	165 (81.2)
	Positive	2	38 (18.8)

Seminal vesicle invasion	No	0	190 (93.5)
	Yes	2	13 (6.5)

Gleason score	2–6	0	120 (59.2)
	3 + 4	1	41 (20.2)
	4 + 3	2	15 (7.3)
	8–10	3	27 (13.3)

Extracapsular extension	Absent	0	162 (79.8)
	Present	1	41 (20.2)

Lymph node invasion	Negative	0	197 (97)
	Positive	1	6 (3)

**Table 2:** Determination of risk groups according to CAPRA-S score.

Risk groups	CAPRA-S each score group	Number (%)	Total *n* (%)
Low risk	0	39 (19.2)	131 (64.5)
	1	51 (25.1)	
	2	41 (20.1)	

Intermediate risk	3	18 (8.8)	52 (25.6)
	4	19 (9.3)	
	5	15 (7.3)	

High risk	6	9 (4.4)	20 (9.9)
	7	2 (0.9)	
	8	2 (0.9)	
	≥9	7 (3.5)	

**Table 3:** C-indexes of CAPRA-S groups and three-risk level model for 3 years BCR-free survival.

Time	Variables	c-index (95% CI)	*p* value
3 years	CAPRA-S score group	0.82 (0.68–0.90)	<0.05
	Three-risk level model	0.78 (0.65–0.88)	<0.05

**Table 4:** Cox proportional hazard model of biochemical recurrence using variables of CAPRA-S score.

Data	*n* (%) BCR+/total	Hazard ratio	95% confidence interval	*p* value
SMI+	18/38 (47.3)	1.60	1.04–2.02	0.035
SVI+	8/13 (62)	1.49	0.95–1.91	0.041
ECE+	13/41 (31.7)	1.12	0.42–1.51	0.102
LNI+	2/6 (33)	0.98	0.44–1.12	0.61

SMI+: surgical margin invasion, SVI+: seminal vesicle invasion, ECE+: extracapsular extension, LNI+: lymph node invasion, and BCR+: biochemical recurrence.
